# Fetal MRI versus postnatal imaging in the MR-compatible incubator

**DOI:** 10.1007/s11547-016-0649-y

**Published:** 2016-06-02

**Authors:** Monika Bekiesinska-Figatowska, Anna Romaniuk-Doroszewska, Agnieszka Duczkowska, Marek Duczkowski, Beata Iwanowska, Sylwia Szkudlińska-Pawlak

**Affiliations:** Department of Diagnostic Imaging, Institute of Mother and Child, ul. Kasprzaka 17a, 01-211 Warsaw, Poland

**Keywords:** Fetal MRI, Prenatal diagnosis, Neonatal MRI, MR-compatible incubator, Postnatal diagnosis

## Abstract

**Introduction:**

One of the aims of fetal magnetic resonance imaging (MRI) is to avoid postnatal scanning. However, clinicians sometimes wish to have postnatal confirmation of prenatal findings. This study’s purpose was to check whether there was indeed the added value of neonatal MRI performed in the MR-compatible incubator (INC) after fetal examination.

**Materials and methods:**

Material consists of 25 neonates (14 girls) who underwent prenatal and postnatal MRI in a 1.5 T scanner, the latter in INC. Mean time of prenatal MRI was 30th gestational week, of postnatal MRI—16th day of life.

**Results:**

In 14 cases (56 %) postnatal findings were the same as prenatal ones. In 11 (44 %) postnatal MRI showed some different/new/more precise results, in two the differences were attributed to other factors than the advantage of postnatal MRI over prenatal one. Altogether then postnatal results were partly discordant with prenatal ones in 9/25 cases (36 %).

**Conclusions:**

In most cases there was no added value of postnatal MRI as compared to prenatal one. This value lied in small details that could not have been noticed on prenatal MRI or required contrast medium administration to be noticed. On the other hand, MR examination performed with use of the dedicated neonatal coils in the MR-compatible incubator is a safe and reliable method of visualization of these small details with better spatial resolution thus helping to establish final diagnosis, treatment plan and prognosis.

## Introduction

One of the aims of fetal magnetic resonance imaging (MRI), especially if it is performed close to term, is to avoid postnatal scanning as it has been documented that in many disorders prenatal MRI can establish the diagnosis and help in the planning of postnatal management, obviating the need for early postnatal imaging [1–3]. However, clinicians in some cases still wish to have postnatal confirmation of prenatal MRI findings. Although the mother’s uterus is a safe “incubator” for the sick fetus, currently we have a secure equivalent of it for a newborn which is the MR-compatible incubator (INC), offering the safe environment during MRI procedure even for unstable neonates [4, 5]. The literature concerning early postnatal MRI in the INC is very scant and devoted to the brain in vast majority of publications. Thanks to the dedicated neonatal coils built in our INC (eight-channel phase-array head coil and whole body twelve-channel phase-array coil consisting of two elements: eight-channel part integrated with the incubator bed and four-channel separate surface coil) we are able to examine not only the neonatal brain but also other organs and systems.

To the best of our knowledge there are no papers in the literature comparing the value of prenatal and postnatal MRI, with the latter performed in this most up-to date equipment. The purpose of this study was to check whether there was indeed the added value of early postnatal MRI performed in the MR-compatible incubator after the fetal examination without the limitation to the head—with respect to the pathology of the whole body.

## Materials and methods

The material consists of 27 neonates who underwent pre- and early postnatal MRI in a 1.5 T scanner. Two neonates could not be examined in the incubator due to the size of extracranial abnormality (suboccipital encephalomeningocele and a tongue tumor). Therefore, finally we included 25 babies (14 girls, 11 boys). Prenatal MRI was performed between the gestational ages of 19 and 38 weeks (mean in the 30th gestational week, GW), postnatal MRI between day 1 (day of birth) and the age of 3 months (mean in the 16th day of life). In four cases two prenatal MR examinations were performed.

Prenatal MRI consisted of the following sequences: single-shot fast spin echo/T2-weighted images (SSFSE/T2WI) in axial, sagittal and coronal planes, fast imaging employing steady state acquisition (FIESTA/2D) in three planes as well, fast spin echo/T1WI (FSE/T1WI), gradient echo echo planar imaging (GRE EPI), susceptibility-weighted imaging (SWI) and diffusion-weighted imaging (DWI) in chosen planes, adequate to the diagnostic problem. The parameters used in the prenatal protocol are presented in Table 1.

**Table 1:** Sequence parameters used in prenatal MRI

Sequence	TR (ms)	TE (ms)	FOV (cm)	MX	ST/IG (mm)	NEX	Others
SSFSE/T2	5000	141.5	44 × 44	320 × 224	3.0/0.3	0.57	
FIESTA/2D	4.2	1.9	44 × 44	224 × 320	3.9/0.3	1	
DWI	7675	85.5	44 × 44	128 × 128	3.0/0.3	5	*b* = 0 and 700
SWI	6750	40	26 × 26	256 × 492	3.0/0.3	4	
GRE EPI	3950	50	44	160 × 160	3.0/0.3	8	
FSE/T1	400	7.8	34 × 34	256 × 160	4.0/1.0	1	

*TR* repetition time, *TE* echo time, *FOV* field of view, *MX* acquisition matrix, *ST/IG* slice thickness/interslice gap, *NEX* number of excitations

Sixteen babies were born at term, nine—preterm, at the gestational age between 31 and 37 weeks (mean in the 34th GW). In all cases included, the study was performed with use of a Nomag IC 1.5 incubator, equipped with three coils: an eight-channel, phased-array head coil and a twelve channel phased-array coil for the whole body, consisting of an eight-channel coil integrated in the incubator and a separate four-channel surface coil.

In postnatal scans, the same sequences were used, and additionally, depending on region of interest (head or body), fluid attenuated inversion recovery (FLAIR) and short TI inversion recovery (STIR) were included in the protocol. The parameters used are shown in Table 2.

**Table 2:** Sequence parameters used in postnatal brain MRI

Sequence	TR (ms)	TE (ms)	FOV (cm)	MX	ST/IG (mm)	NEX	Others
SE/T1	480	11	18 × 13.5	256 × 192	3.0/0.3	2	
FSE/T2	4500	83.8	18 × 14.4	384 × 224	3.0/0.3	2	
FLAIR	8000	148.6	18 × 18	320 × 192	3.0/0.3	1	TI = 2000
DWI	5200	99.9	18 × 18	128 × 128	3.0/0.3	2	*b* = 0 and 1000
SWI	6000	40	18 × 18	256 × 512	3.0/0.3	4	

*TR* repetition time, *TE* echo time, *FOV* field of view, *MX* acquisition matrix, *ST/IG* slice thickness/interslice gap, *NEX* number of excitations, *TI* inversion time

## Results

In 14 cases (56 %) the MRI findings obtained after birth were the same as in the prenatal examination(s). These results are presented in Table 3. In the remaining 11 cases (44 %) postnatal MRI showed some different or new or more precise results as compared to the fetal one: subependymal heterotopia, callosal hypoplasia, agenesis of the anterior commissure, optic nerves hypoplasia and cranial nerves V, VII, VIII emerging together from the brainstem, cleft palate, subcutaneous hemangioma and not the intraosseous one, atretic encephalocele, unicornuate uterus, multiple enhancing nodules as an addition to the enormous cystic tumor in the abdomen and pelvis in a case of blue rubber bleb nevus syndrome (BRBNS). In 1 baby out of these 11 postnatal MRI performed at term equivalent revealed normal brain gyration while at the gestational age of 26 weeks it was delayed by approximately 2 weeks—so it showed normal brain maturation which has been inharmonious earlier rather than the advantage of postnatal MRI over prenatal one. In another baby with clinically evident microcephaly, cerebellar hypoplasia that was diagnosed prenatally was not confirmed as such on postnatal MRI which revealed proportionally small cerebrum and cerebellum. Altogether then the results of postnatal MRI were partly discordant with prenatal one in 9/25 cases which accounts for 36 % of all the cases. Besides in two cases there were additional postnatal findings related most likely to perinatal insult, such as subependymal bleeding and ischemic focus in the brain. The discrepancies between prenatal and postnatal examinations are shown in Table 4.

**Table 3:** Concordant diagnoses from fetal and postnatal MRI in the analyzed group

Case no.	Age at fetal MRI (GW)	Age at postnatal MRI/corrected age	Results of fetal MRI	Results of postnatal MRI
2	31	3 days/36 GW	Hydrocephalus, aqueductal stenosis, rhombencephalosynapsis	Hydrocephalus, aqueductal stenosis, rhombencephalosynapsis
4	33	10 days/mature	ACC, interhemispheric cyst, neuronal migration disorder in right cerebral hemisphere	ACC, interhemispheric cyst, polimicrogyria and heterotopia in right cerebral hemisphere
5	34	11 days/mature	Abdominal cyst	Duplication cyst or mesenteric cyst
6	31	3 days/mature	Asymmetric VM (*L* > *R*), most likely posthemorrhagic	Asymmetric posthemorrhagic VM (*L* > *R*)
7	32	6 days/mature	Normal brain	Ischemic focus related most likely to perinatal insult; otherwise normal brain
8	35	1 day/mature	Hypoplastic CC, pericallosal lipoma	Hypoplastic CC, pericallosal lipoma
10	34	12 days/mature	Normal brain	Normal brain
12	29	1 day/31 GW	Face tumor, without fat, with hemorrhage	Face tumor, without fat, with hemorrhage
13	32	1 day/36 GW	Dandy-Walker variant	Dandy-Walker variant
15	35	1 day/mature	Unilateral microphtalmia, cleft lip and palate	Microphtalmia, cleft lip and palate on the left
16	28	0 days/mature	Transsphenoidal encephalocele	Transsphenoidal encephalocele
19	33	2 months/44 GW	ACC	ACC
21	34	1 day/mature	Neck cyst	Neck cyst
25	35	5 weeks/42 GW	ChIIM with MMC in Th-S and tethered cord, ectopic left kidney	ChIIM with MMC in Th-S and tethered cord (after surgery), ectopic left kidney

*ACC* agenesis of the corpus callosum, *VM* ventriculomegaly, *CC* corpus callosum, *ChIIM* Chiari II malformation, *MMC* myelomeningocele, *Th-S* from thoracic to sacral part of vertebral column

**Table 4:** Discordant diagnoses in fetal and postnatal MRI in the analyzed group

Case no.	Age at fetal MRI (GW)	Age at postnatal MRI/corrected age	Results of fetal MRI	Results of postnatal MRI
1	22 and 31	1 day/mature	MMC, scoliosis, single kidney	MMC, scoliosis, single kidney, **unicornuate uterus**
3	21 and 33	1 day/35 GW	Encephalocele containing CSF	Encephalocele containing CSF **and some dysplastic tissue, subependymal heterotopia**
*9*	*26*	*2* *months/41* *GW*	*Gyration delayed by appr. 2* *weeks*	***Normal gyration***
11	25	1 day/31 GW	Huge cyst in abdomen and pelvis	Huge cystic tumor, **numerous enhancing lesions in liver, diaphragm and chest wall**
14	34	5 days/mature	Cleft lip and palate, eye bulbs deformation	Cleft lip and palate, bilateral coloboma, **hypoplastic optic nerves, nerves V, VII, VIII emerging together from brainstem, CC hypoplasia**
17	20	0 day/32 GW	Holoprosencephaly	Holoprosencephaly, **subependymal heterotopia**
18	38	2 days/mature	Hemangioma intraosseous or subcutaneous on the head	**Subcutaneous** hemangioma
*20*	*27 and 37*	*3* *months/mature*	*Hypoplastic cerebellum*	***Microcephaly***
22	34	2 days/mature	Pericallosal lipoma, dacryocystocele	Pericallosal lipoma, **CC hypoplasia**, dacryocystocele, **nodule in the mouth,** **unilateral alveolar cleft**
23	36	17 days/mature	ACC, abnormal gyration	ACC, polymicrogyria, **bilateral cleft palate**
24	19 and 25	1 day/mature	Fluid-filled lesion in soft tissues of the head	**Atretic encephalocele, fetal configuration of straight sinus**

Differences between pre- and postnatal diagnosis (new postnatal findings) are shown in bold

Cases of inharmonious development rather that of the real advantage of postnatal MRI over prenatal one are shown in italics

*MMC* myelomeningocele, *CSF* cerebrospinal fluid, *CC* corpus callosum, *ACC* agenesis of the corpus callosum

## Discussion

In more than half of our patients the diagnosis was fully and properly established on prenatal MR examination and only confirmed postnatally. These diagnoses included ventriculomegaly/hydrocephalus, aqueductal stenosis, rhombencephalosynapsis, cerebellar hypoplasia, callosal agenesis/hypoplasia, interhemispheric cyst, pericallosal lipoma, gray matter heterotopia, transsphenoidal encephalocele, face tumor, microphtalmia, cleft lip and palate, neck cyst, Chiari II malformation, ectopic kidney and abdominal cyst.

**Fig. 1:**
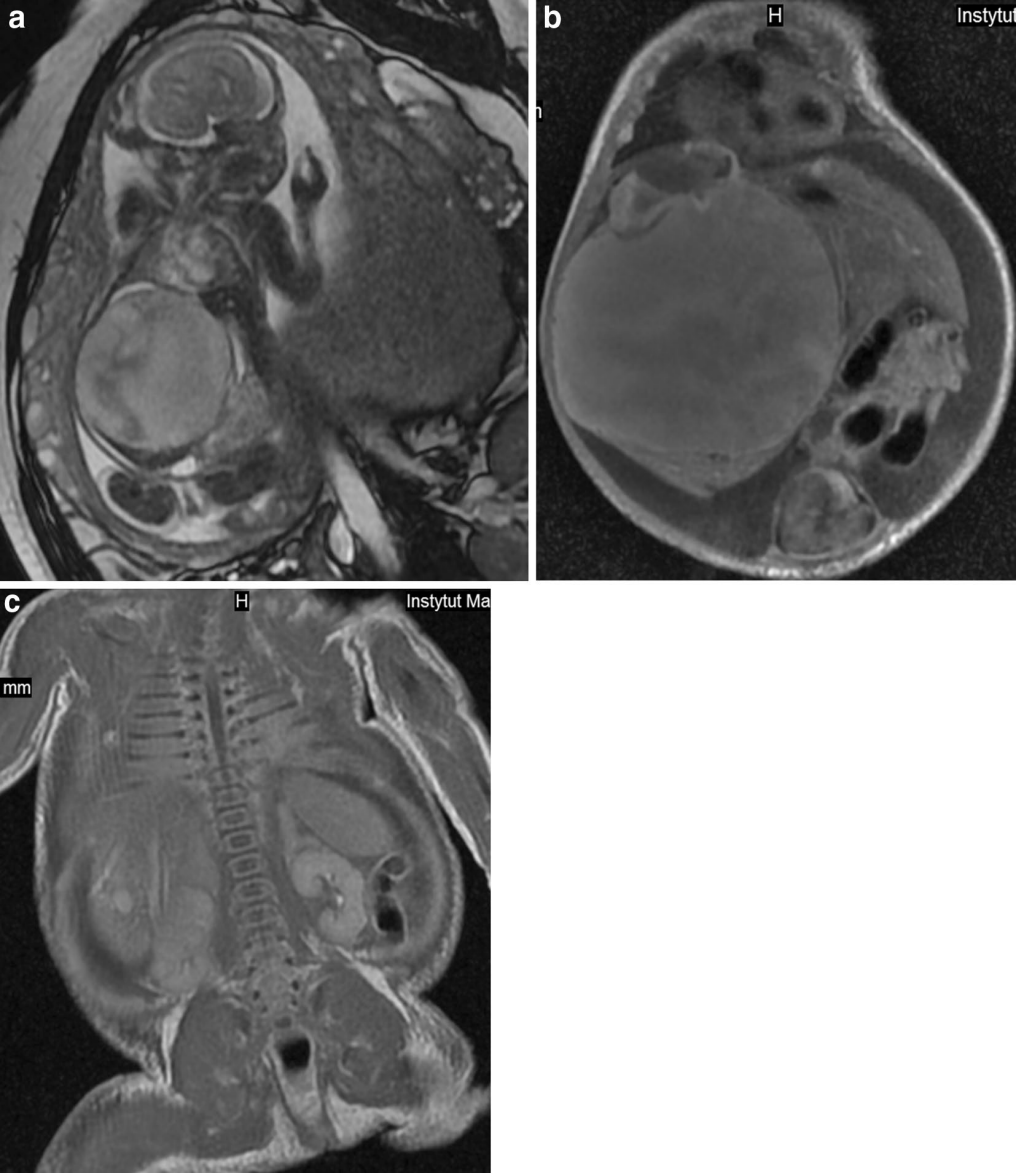
Case no. 11. **a** Fetal MRI. Huge abdominal and pelvic cyst. **b** Postnatal MRI. The dominant cyst and a small enhanced nodule in the *left* lobe of the liver. **c** Postnatal MRI. Enhanced nodules in the *right* lobe of the liver behind the main mass, in the chest wall on the *right side* and in the diaphragm on the *left*

Several studies compared the results of prenatal MRI with postnatal one. Most of them concerned malformations of the central nervous system (CNS) [e.g. 2,6–8] although other systems have also been described [9]. In our material there are also studies of other organs and systems: vertebral column and spinal canal in one case, head and neck in three cases, and abdomen and/or pelvis in three, which accounts for 28 % of the study group. This makes the material inhomogeneous but covers the whole spectrum of MRI utility in both prenatal and postnatal studies. Although congenital abnormalities/damage to CNS still remain the main indication in case of neonatal MR examinations, there is a growing role of this method in body imaging. The technical progress and development of dedicated neonatal body coils which vendors had started to build in the INC allowed evaluation of other parts of the body and not only the brain which is the only organ that has been described in previous studies. Regardless of which organ or system they relate to, prenatal MR examinations allow early planning of postnatal interventions, speeding up the postnatal diagnostic process and minimizing delay in treatment after delivery. In many cases treatment can readily be based on the information available from prenatal imaging [2]. Although fetal MRI has gone far beyond T2-weighted imaging alone and numerous other sequences are available nowadays [10], it still does not have the same diagnostic quality and variety of pulse sequences as are available in the postnatal examination. The use of gadolinium-based contrast media is also reserved for postnatal MRI. Therefore, for example in oncological cases, the clinicians wish to have accurate information about the origin of the tumor, the invasion of adjacent organs, and the presence or absence of metastases after birth. In our case no. 11, we saw prenatally the huge abdominal cyst (Fig. 1a) but only after birth we could appreciate numerous tiny nodules in other localizations that enhanced strongly with contrast medium (Fig. 1b,c). We diagnosed this case as a malignant tumor with metastatic spread but we were wrong as these turned to be venous malformations in BRBNS.

**Fig. 2:**
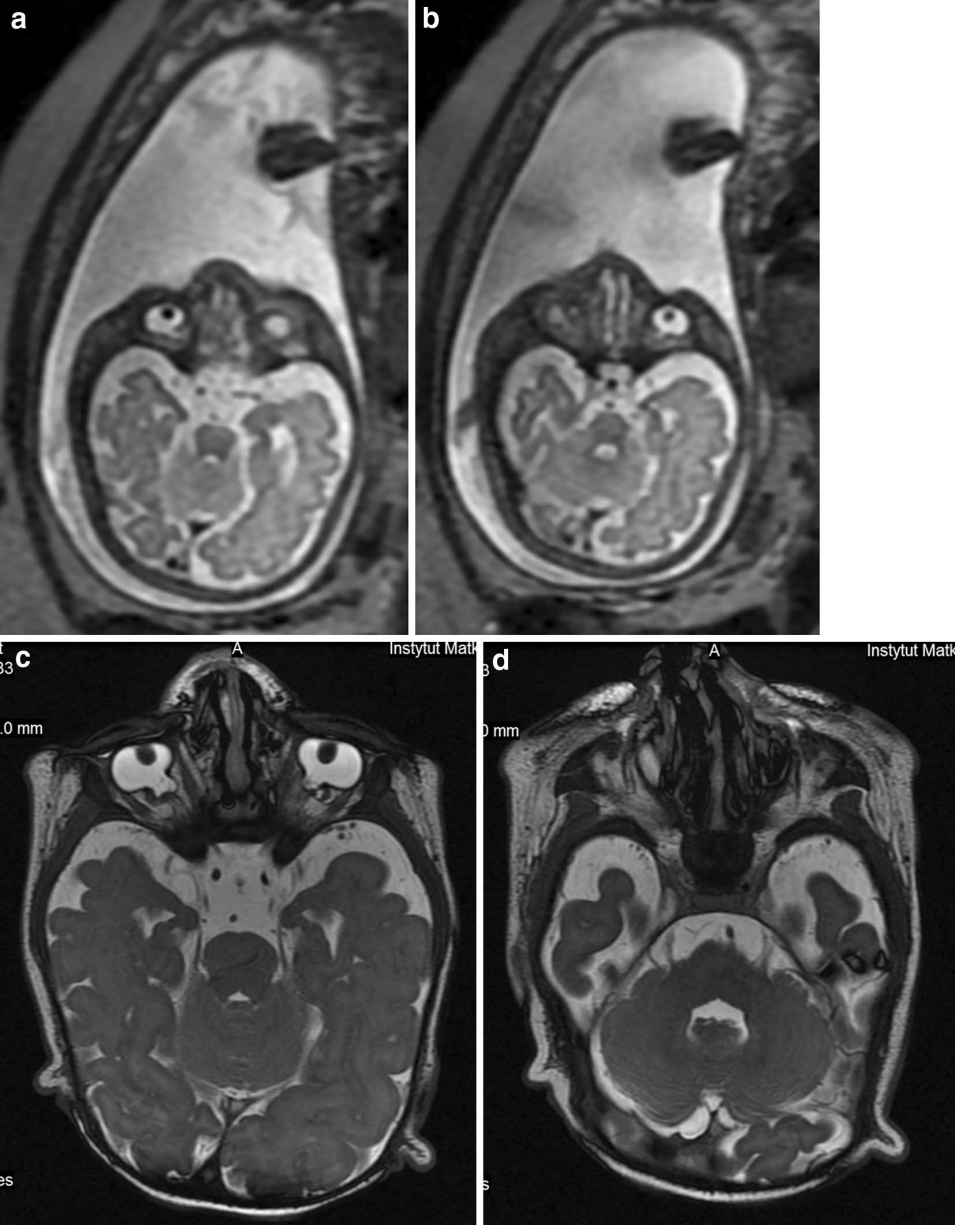
Case no. 14. **a**,**b** Fetal MRI. T2-weighted images, axial plane. Only deformation of the eye bulbs can be appreciated on these images. **c** Postnatal MRI. T2-weighted image, axial plane. Bilateral coloboma. **d** Postnatal MRI. T2-weighted image, axial plane. On the *left side* cranial nerves V, VII and VIII emerge together from the brain stem. On the *right* only cranial nerve V is visible in this section

Tiny structures are in general difficult to see on prenatal MRI. They are best seen if changed in a cystic way, due to T2-hyperintensity. Dacryocystocele is a good example. Cranial nerves are in turn a good example of tiny structures which are impossible to assess on prenatal MRI with the exception of the optic nerves which are visible in most fetuses in the second half of pregnancy—in our experience earlier than reported in the literature: according to Brugger, visualization of the optic nerve is associated with change of signal intensity of the retrobulbar adipose tissue, which occurs in the third trimester of pregnancy [11], i.e., since 27th GW. It does not mean, however, that we are able to evaluate whether optic nerves are normal or hypoplastic. So hypoplastic optic nerves and cranial nerves V, VII and VIII emerging together from the brainstem were absolutely impossible to be assessed as such on prenatal MRI in our case no. 14 (Fig. 2a–d). Searching in PubMed for “fetus, cranial nerves, MRI” we have not found any reports concerning fetal MRI in vivo.

**Fig. 3:**
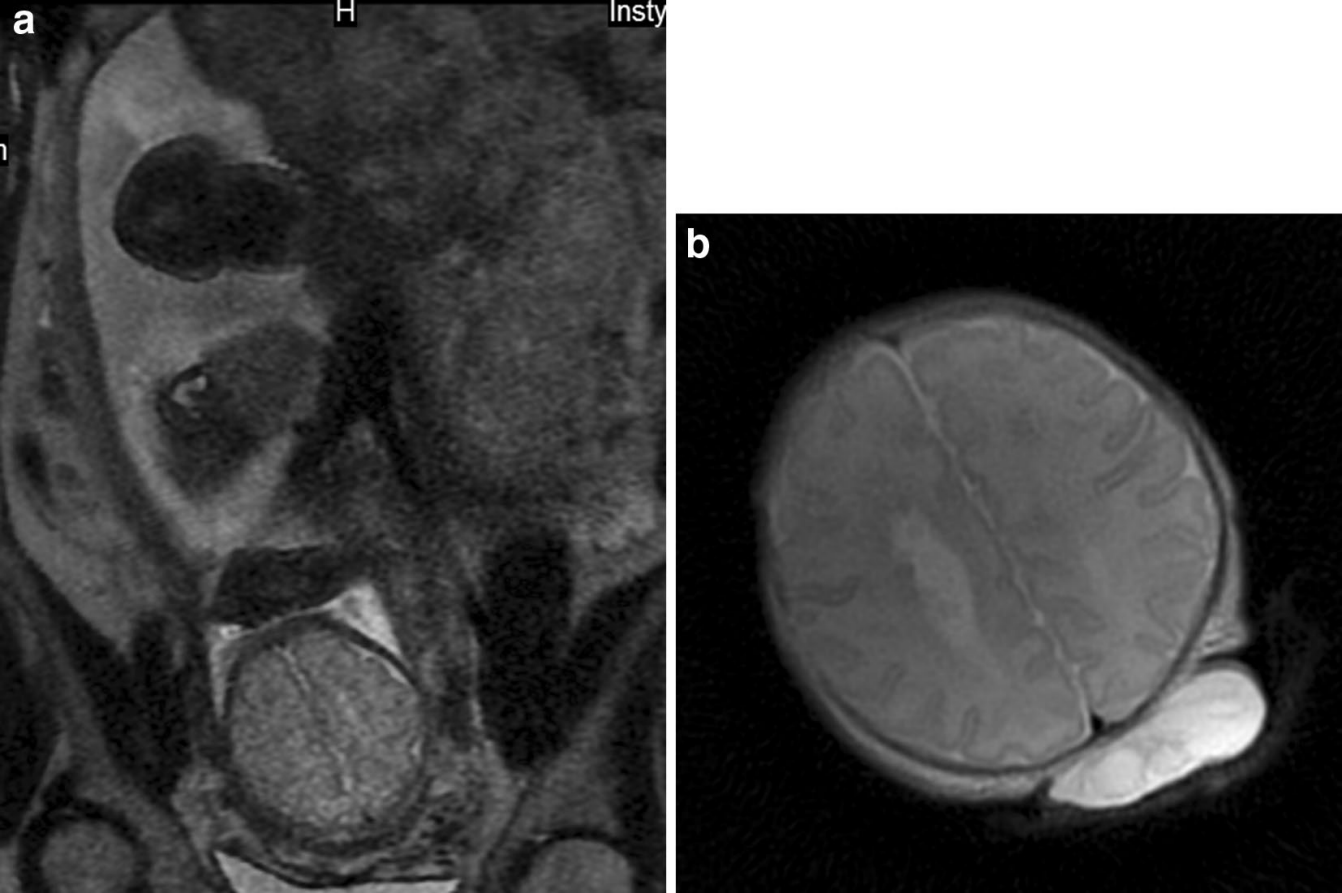
Case no. 3. **a** Prenatal MRI. T2-weighted image, axial plane. *Gray* *matter* heterotopia is not visible. **b** Postnatal MRI. T2-weighted image, axial plane. *Gray* *matter* hetrotopia was diagnosed only after birth. Encephalocele is also seen in this section

Also subependymal gray matter heterotopia belongs to the list of tiny elements that are difficult to appreciate on prenatal MRI. In our material, it was missed in two cases: no. 3 and 17 (Fig. 3a–b). This is in line with the results of other authors who explain the difficulty in identifying subependymal heterotopia with the fact the nodules have small size and signal intensity similar to the adjacent germinal matrix [8].

If the corpus callosum of the fetus is only hypoplastic, the typical MRI symptoms indicating its agenesis, such as enlargement of the ventricular atria and the occipital horns (colpocephaly), marked separation of the bodies of the lateral ventricles, laterally positioned frontal horns with concave medial borders, elevation of the third ventricle and radial disposition of the sulci on the internal aspects of the hemispheres, may be absent [12]. In our material, this was the case in two fetuses: nos. 14 and 22, and we missed the diagnosis of callosal hypoplasia in these fetuses.

**Fig. 4:**
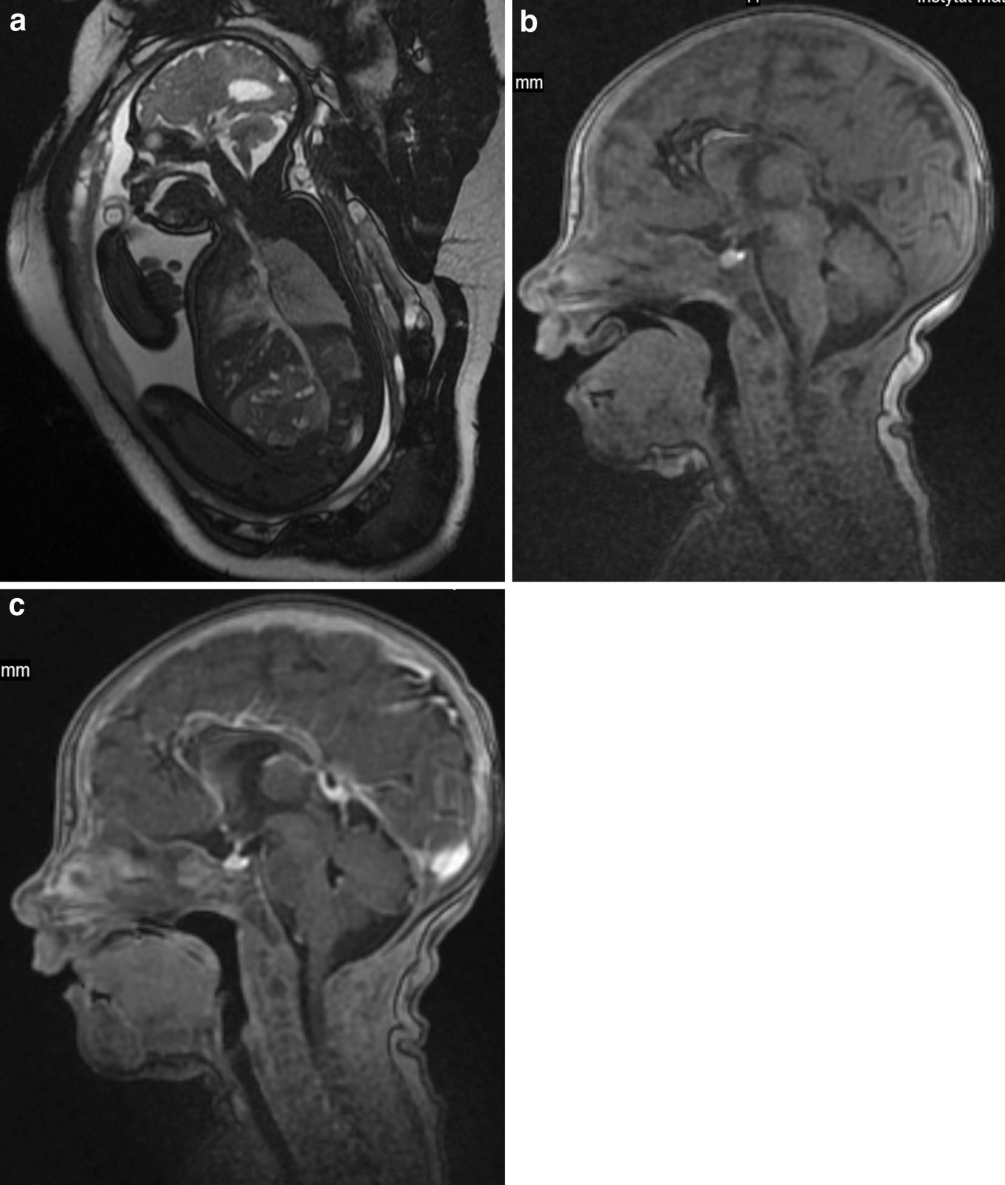
Case no. 22. **a** Fetal MRI. FIESTA/2D image, sagittal plane. The irregular contour of the palate is visible but impossible to assess as a tumor. **b** Postnatal MRI. 3D/T1-weighted image. The pedunculated nodule arising from the anterior part of the palate is well seen in this midsagittal section with the open mouth of the newborn. **c** Postnatal MRI. 3D/T1-weighted image after gadolinium injection, sagittal plane. With the mouth closed the nodule is no longer visible. One can only state that it does not enhance

Usually, if we deal with clefts of the primary palate it means cleft lip and alveolus which is diagnosed properly on fetal MRI [13]. In our case no. 22, we diagnosed cleft alveolus on postnatal MRI which was confirmed during surgery. In the literature, we did not find a report on isolated alveolar cleft without cleft lip. The baby was operated on due to the pedunculated fibroma in the mouth with dimensions of 15 × 7 × 7 mm after birth, which was not diagnosed on prenatal MRI either (Fig. 4a–c). It was hard to see postnatally when the newborn kept the mouth closed but with the open mouth, it was easy to appreciate and measure. The small size of the lesion explains why it was not detected on prenatal MRI. Also, isolated cleft palate without cleft lip is difficult to diagnose although MRI is more helpful in this aspect than sonography in which shadowing from the surrounding facial bones is very disturbing. However, MR images are also disturbed by fetal motion between and during image acquisitions [14] and we also appreciated isolated bilateral cleft palate as late as in postnatal examination.

As stated above it is not always easy to find the place of origin of the lesion on prenatal MRI. In case no. 18, we saw a hemangioma on the fetus’ head but in some sequences and planes it seemed to be intraosseous while in the others—subcutaneous. Postnatal MRI confirmed its superficial localization. In case no. 24, we did not see the connection between the inside of the skull and a small fluid-filled lesion beneath the skin of the head and, therefore, we did not establish the diagnosis of atretic encephalocele on prenatal MRI performed early, at 25 GW.

**Fig. 5:**
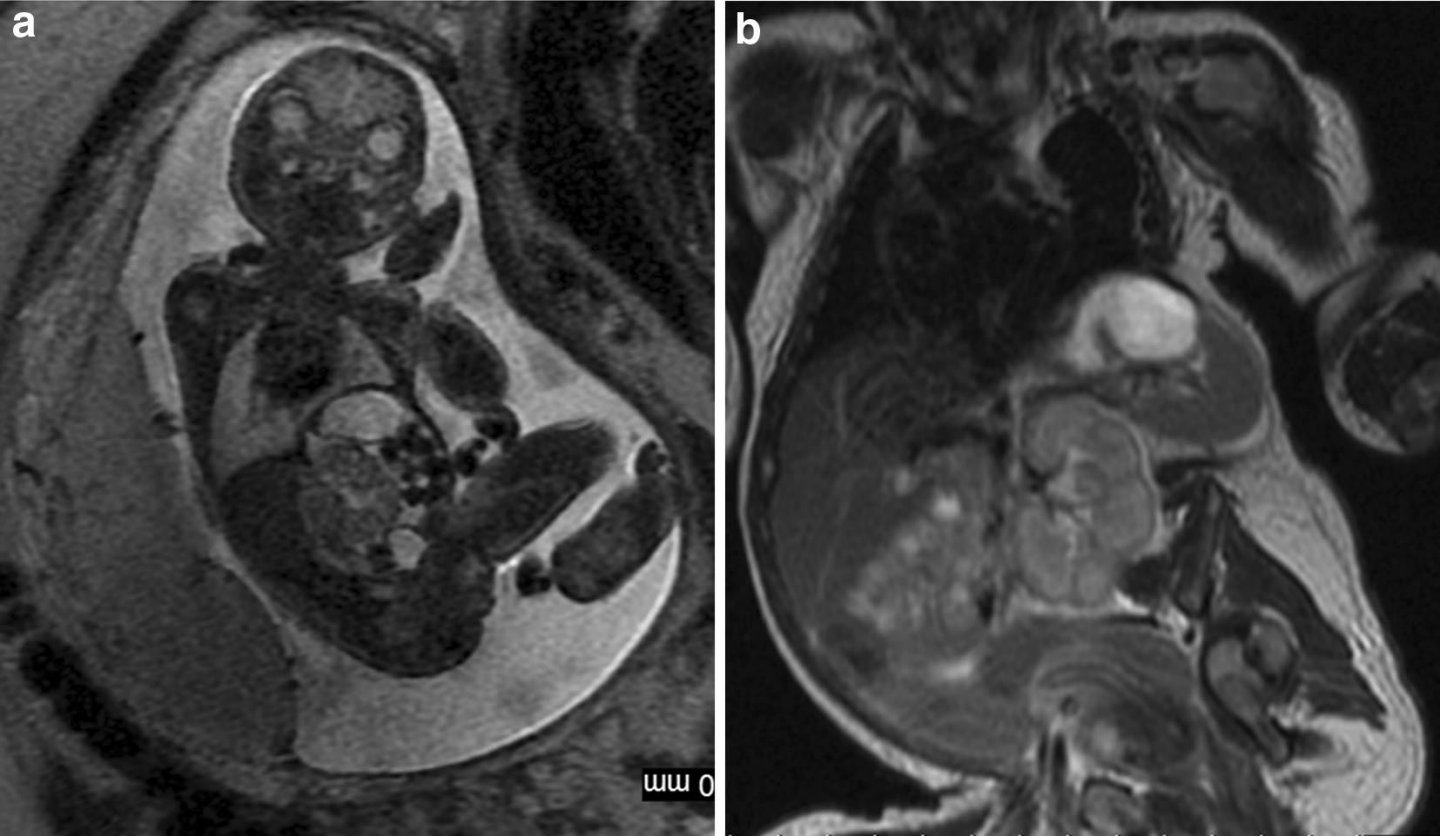
Case no. 1. Single ectopic kidney is seen in both examinations. **a** Prenatal MRI. T2-weighted image, coronal oblique plane. The uterus was impossible to assess. **b** Postnatal MRI. T2-weighted image, coronal plane. Unicornuate uterus with a typical *banana shape*

Finally in case no. 1 on postnatal MRI, we found uterine anomaly—unicornuate uterus. On prenatal examination uterus can be identified as a tiny structure between the urine-filled T2-hyperintense, T1-hypointense bladder and meconium-filled rectum which is T2-hypointense and T1-hyperintense at this gestational age (31 weeks). Uterine cavity can be seen only if it is filled with fluid as in case of hydrometrocolpos, otherwise it is invisible and impossible to assess (Fig. 5a). So any abnormal shape of the cavity can be diagnosed only after birth (Fig. 5b) [15].

We still face the problem with spatial resolution in fetal MRI. Postnatal MRI is not free of this problem but is definitely less affected by it. With the advent of two-dimensional fast imaging employing steady-state acquisition (FIESTA/2D) technique with parallel imaging, which was also used in this study, prenatal MRI achieved temporal resolution at less than half a second and quite high spatial resolution [16], however, it is still lower than that of neonatal MRI. By definition then, the number of details seen prenatally is limited as compared to postnatal visualization.

A recent study of the human fetus comparing signal-to-noise ratio (SNR) and image quality between 1.5 and 3.0 T MRI has shown higher image resolution and SNR, and—in approximately one-third of cases—superior tissue contrast and conspicuity in a 3.0 T scanner compared to 1.5 T MRI [17]. This might be the future for obtaining better images of fetuses with more anatomical and pathological details but for now most of fetal studies are performed on 1.5 T units and we do not see everything. Fortunately, most of the changes that went unnoticed on prenatal studies in our material had no significant impact on the management of newborn babies at this very moment of their lives. However, we do remember that many of these patients are syndromic ones and that the small undetected details cannot be underestimated as far as a final diagnosis is concerned.

A limitation of our study is that the sample size is relatively small. However, our study group was limited to patients who were referred for clinical fetal MRI first and then for MRI after birth. Continuously improving prenatal MRI and striving not to repeat this study postnatally, we have a limited number of cases in which clinicians consider MRI as indispensable after birth. This limitation could be overcome by performing postnatal MRI on all children who undergo fetal one but it would be unethical to sedate a neonate only for research purposes.

Another limitation is inhomogeneity of the material which consists of both neuroimaging and body imaging. However—in our opinion—being a limitation, it is also an advantage since—as mentioned above—the collected material reflects the whole spectrum of indications for which both prenatal and postnatal MRI is performed at present time.

## Conclusions

The obvious advantage of imaging in the MR-compatible incubator is that a baby that has already been born is diagnosed, and not the tiny fetal body in a mother’s body, which improves image quality and provides opportunity to visualize tiny elements of anatomy as well as tiny pathological lesions, but still in most of our cases there was no added value of postnatal MRI as compared to prenatal one. This value lied in small details that could not have been noticed on prenatal MRI or required contrast medium administration to be noticed. On the other hand, MR examination performed with use of the dedicated neonatal coils in the MR-compatible incubator is a safe and reliable method of visualization of these small details with better spatial resolution thus helping to establish final diagnosis, treatment plan and prognosis.
